# ICTV Virus Taxonomy Profile: Satyavativiridae 2026

**DOI:** 10.1099/jgv.0.002249

**Published:** 2026-04-24

**Authors:** Apoorva Prabhu, Christian Rinke

**Affiliations:** 1 School of Chemistry and Molecular Biosciences, Australian Centre for Ecogenomics, The University of Queensland, Queensland, QLD 4072, Australia; 2 Sydney Institute of Marine Science, Sydney, New South Wales 2088, Australia; 3 Climate Change Cluster (C3), University of Technology Sydney, Sydney, NSW 2007, Australia; 4 Department of Microbiology, University of Innsbruck, 6020 Innsbruck, Austria

**Keywords:** *Adrikavirales*, ICTV Report, marine archaea, *Satyavativiridae*, *Poseidoniales*, taxonomy

## Abstract

Members of the family *Satyavativiridae* are dsDNA viruses associated with the host *Poseidoniales*, a marine archaeal lineage. Genomes assigned to this group have been identified via long-read sequencing of brackish estuarine samples. These viruses represent a novel viral lineage in the order *Adrikavirales* distinct from other *Poseidoniales* viruses known as ‘magroviruses’, a collective term derived from Marine Group II Euryarchaeota (former name of *Poseidoniales*) viruses. The family includes the genus *Vyasavirus* and the species *Vyasavirus brisbanense*. The virus genome is a linear dsDNA of 87 kbp and encodes proteins involved in the morphogenesis of virions, which are predicted to be composed of an icosahedral capsid and helical tail, characteristic of members of the class *Caudoviricetes*. This is a summary of the International Committee on Taxonomy of Viruses Report on the family *Satyavativiridae*, which is available at ictv.global/report/satyavativiridae.

## Virion

**Fig. 1.**
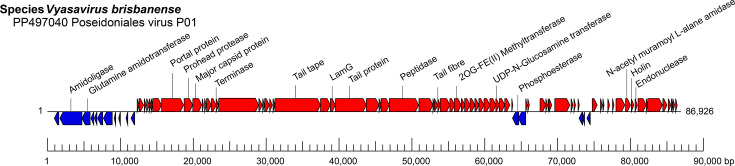
Genome organization of Posedoniales virus P01.

The complete genome of Poseidoniales virus P01 (species *Vyasavirus brisbanense*) includes a morphogenetic module with genes for head and tail proteins, such as major capsid protein that are typical of viruses in the class *Caudoviricetes* (Table 1, Fig. 1). Other morphogenesis proteins include the prohead protease, portal protein and the large subunit of the terminase. In addition, the gene arrangement and size and major capsid protein phylogeny of satyavativirids show similarity to those features of tailed haloviruses (such as viruses in the family *Druskaviridae*), suggesting that they likely possess icosahedral heads and helical tails [1,2].

**Table 1.** Characteristics of members of the family *Satyavativiridae*

Example	Poseidoniales virus P01 (PP497040), species *Vyasavirus brisbanense*
Virion	Predicted icosahedral capsid and helical tail
Genome	Linear dsDNA of 87 kbp
Replication	Unknown, possibly using host replisome
Translation	Unknown
Host range	*Archaea*: *Poseidoniales* (predicted)
Taxonomy	Realm *Duplodnaviria*, kingdom *Heunggongvirae*, phylum *Uroviricota*, class *Caudoviricetes*, order *Adrikavirales*; the family *Satyavativiridae* includes the genus *Vyasavirus* and the species *Vyasavirus brisbanense*

## Genome

The complete genome of Poseidoniales virus P01 (species *Vyasavirus brisbanense*) is a dsDNA molecule of 87 kbp and predicted to encode 85 proteins.

## Replication

Unknown, possibly uses host machinery for DNA replication.

## Taxonomy

**Fig. 2.**
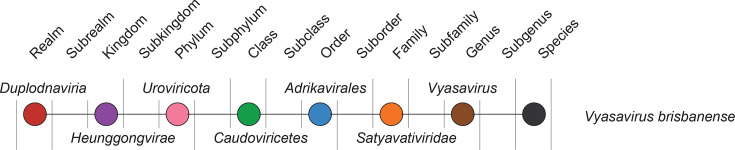
Relationship of taxa connected to the family *Satyavativiridae*.

Current taxonomy: ictv.global/taxonomy. Viruses in the family *Satyavativiridae* represent a novel lineage distinct from magroviruses [3,7] and are instead assigned to the order *Adrikavirales* [7] (Fig. 2). The name *Satyavativiridae* derives from Satyavati, the child of Adrika, who later became a Queen in ancient Indian mythology, while the name *Vyasavirus* comes from Vyasa, the child of Satyavati, a revered sage who compiled the Vedas in ancient Indian mythology. The species epithet *brisbanense* refers to the sampling source, the Brisbane River.

## Resources

Full ICTV Report on the family *Satyavativiridae*: ictv.global/report/satyavativiridae.

## References

[1] Baquero DP, Liu Y, Wang F, Egelman EH, Prangishvili D (2020). Structure and assembly of archaeal viruses. Adv Virus Res.

[2] Liu Y, Demina TA, Roux S, Aiewsakun P, Kazlauskas D (2021). Diversity, taxonomy, and evolution of archaeal viruses of the class *Caudoviricetes*. PLoS Biol.

[3] Nishimura Y, Watai H, Honda T, Mihara T, Omae K (2017). Environmental viral genomes shed new light on virus–host interactions in the ocean. mSphere.

[4] Philosof A, Yutin N, Flores-Uribe J, Sharon I, Koonin EV (2017). Novel abundant oceanic viruses of uncultured marine group II Euryarchaeota. Curr Biol.

[5] Zhou Y, Zhou L, Yan S, Chen L, Krupovic M (2023). Diverse viruses of marine archaea discovered using metagenomics. Environ Microbiol.

[6] Xu B, Fan L, Wang W, Zhu Y, Zhang C (2023). Diversity, distribution, and functional potentials of magroviruses from marine and brackish waters. Front Microbiol.

[7] Prabhu A, Zaugg J, Chan CX, McIlroy SJ, Rinke C (2025). Insights into phylogeny, diversity and functional potential of *Poseidoniales* viruses. Environ Microbiol.

